# Randomized clinical trial of photobiomodulation and glass ionomer sealant for hypersensitivity in molar incisor hypomineralization

**DOI:** 10.1038/s41598-025-17454-8

**Published:** 2025-08-29

**Authors:** Ana Laura Fossati, Amanda Rafaelly Honório Mandetta, Ana Paula Taboada Sobral, Laura Hermida Bruno, Natália Osório Viarengo, Maria Roxana Ferreira Sertaje, Elaine Marcílio Santos, Marcela Letícia Leal Gonçalves, Raquel Agnelli Mesquita-Ferrari, Kristianne Porta Santos Fernandes, Anna Carolina Ratto Tempestini Horliana, Lara Jansiski Motta, Alessandro Melo Deana, Lourdes Aparecida Martins dos Santos-Pinto, Sandra Kalil Bussadori

**Affiliations:** 1 Interinstitutional Doctorate, Universidade Católica do Uruguai, Montevideo, Uruguay; 2 Postgraduation Program in Biophotonic Medicine, Universidade Nove de Julho, UNINOVE, Rua Vergueiro, 235/249 - Liberdade, São Paulo, SP 01504-000 Brazil; 3 Postgraduation Program in Health and Environment, Universidade Metropolitana de Santos, Santos, SP Brazil; 4 Research of Department of Pediatric Dentistry, São Paulo State University, Araraquara School of Dentistry, UNESP – Araraquara, São Paulo, Brazil

**Keywords:** Complementary medicine, Photobiomodulation, Pediatrics, Pain management, MIH, Signs and symptoms, Optics and photonics

## Abstract

This study evaluated the impact of photobiomodulation (PBM) combined with glass ionomer sealant on hypersensitivity, oral hygiene, and sealant retention in molars affected by molar incisor hypomineralization (MIH). Forty-nine children (6–12 years) with MIH (Grade 3, 4a, or 4b) per MIH-TNI criteria were randomly assigned to two groups: control (n = 25, fluoride toothpaste, sealant with self-curing glass ionomer cement, simulated PBM) and treatment (n = 24, fluoride toothpaste, sealant with self-curing glass ionomer cement, active PBM). Hypersensitivity (SCASS, VAS), oral hygiene (OHI-S), and sealant retention (CCC system) were assessed. Forty-six children completed the study and were evaluated. At all post-baseline time points, the treatment group showed significantly lower VAS and SCASS scores (*p* < 0.05). OHI-S improved in both groups, with a positive correlation between reduced hypersensitivity and better oral hygiene. Sealant retention was higher in the treatment group after 30 days, with 56.5% of teeth showing complete retention versus 17.4% in the control group (*p* = 0.002). In the control group, greater hypersensitivity correlated with worse retention. PBM combined with glass ionomer cement reduces hypersensitivity, improves oral hygiene, and enhances sealant retention in MIH-affected molars. The combined treatment provides superior results in hypersensitivity reduction and sealant retention compared to sealant alone, offering a more effective approach for managing MIH.

*Trial registration*: NCT05370417 in ClinicalTrials.gov

**Supplementary Information:**

The online version contains supplementary material available at 10.1038/s41598-025-17454-8.

## Introduction

Molar incisor hypomineralization (MIH) is characterized by qualitative enamel defects during the mineralization phase of the teeth. This is a multifactorial condition with systemic origins and a genetic component that affects at least one of the permanent first molars and may affect the permanent incisors^1,2^. The clinical characteristics are creamy-white or brownish yellow demarcated opacities that vary depending on the severity of the condition. The global prevalence of MIH is 14.2%, with variations depending on the geographic region studied^3^.

Dentinal hypersensitivity constitutes another characteristic of MIH. Although more frequent in severely affected teeth, this condition can also be seen in teeth without post-eruptive fractures^4^.

Dentin hypersensitivity can manifest during daily activities such as consuming hot or cold foods or beverages, brushing teeth, or spontaneously. This condition is often exacerbated in teeth with MIH, where the enamel is more porous and less resistant, increasing the vulnerability to dentin hypersensitivity. MIH can result in significant discomfort and impact the quality of life of patients^5^.

Photobiomodulation (PBM) with low-power laser does not have any known adverse effects. Additionally, it promotes an analgesic effect by inducing neural changes^6^. Pain relief occurs through the stimulation of nerve cells, changes in membrane potential, and a decrease in neuron excitability. Furthermore, it is responsible for promoting blood circulation, cell regeneration, and inflammation modulation^7,8^.

According to a systematic review aimed at evaluating the effectiveness of PBM in the treatment and prevention of dentin hypersensitivity in teeth without MIH, PBM treatment showed the same effectiveness as desensitizing agents, and the best results were found with the combination of treatment protocols^9^.

The first study on PBM as a complement to treatment with fluoride varnish for the reduction in hypersensitivity in teeth with MIH revealed that laser treatment led to a better immediate result in comparison to the application of high-concentration fluoride. However, long-term results were similar in the comparison of fluoride alone and fluoride combined with laser therapy^10^.

Glass ionomer sealants are recognized as an effective strategy for reducing hypersensitivity in molars affected by MIH^11^. Considering the capacity of this material to adhere to dental tissues with structural defects and the successful use of laser in the cervical region of restorations for the reduction in dentinal hypersensitivity reported in previous studies, our hypothesis is that treatment with glass ionomer sealant combined with PBM therapy in patients with MIH and hypersensitivity is effective at reducing pain, thus enabling the brushing of affected teeth.

Therefore, the primary aim of the present study was to determine the effect of PBM combined with a glass ionomer sealant on hypersensitivity in molars with MIH. Additionally, oral hygiene and sealant retention were evaluated as secondary outcomes.

## Methods

**Fig. 1:**
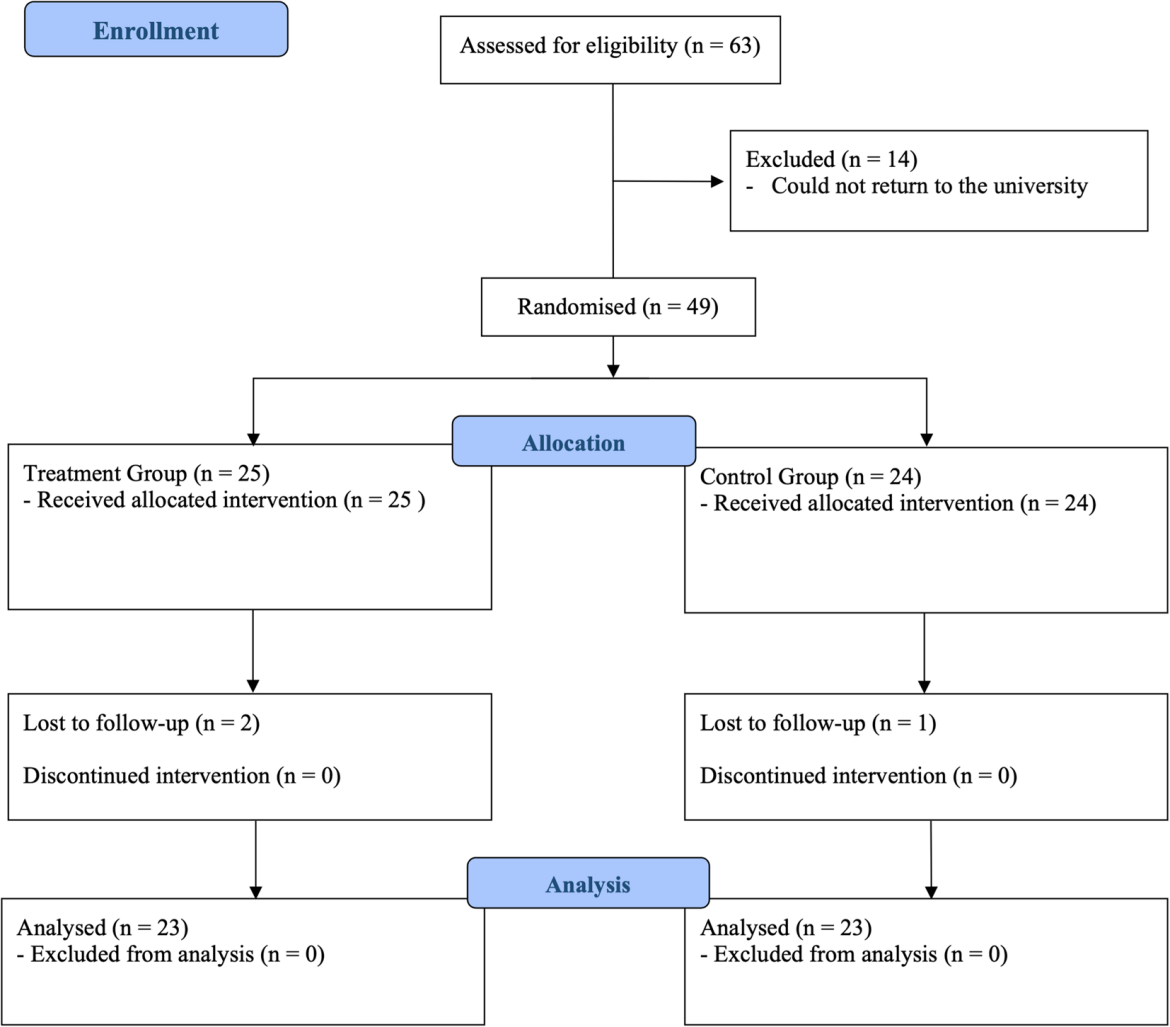
CONSORT 2010 flow diagram.

A randomized, controlled, blind, clinical trial was conducted. The protocol was published in June 2023 and was prospectively registered at ClinicalTrials.gov (Identifier: NCT06692257). The study was conducted with adjustments in sample size calculation and statistical analysis to enhance methodological rigor, as detailed in the sample size calculation section^12^. The Consolidated Standards of Reporting Trials (CONSORT)^13^ were followed to ensure high research standards and transparency. Figure 1 displays the flow diagram of the study.

### Description of sample

Forty-nine female and male patients were recruited from the internal and external clinics of the School of Health Sciences of *Universidade Católica do Uruguai* as well as a private dental office. Each patient received treatment on a single tooth to avoid a systemic effect of PBM that could influence the end result. The recruitment period for this study took place from July 19, 2022, to March 12, 2024.

### Ethical approval

This study received approval from the Human Research Ethics Committee of Universidad Católica del Uruguay "Dámaso Antonio Larrañaga" (certificate number: CEUCU 220516) and was registered in ClinicalTrials.gov (trial registration number: NCT05370417, First Posted: 11/05/2022). After receiving verbal and written clarifications about the study, the legal guardians agreed to their children’s participation by signing a written informed consent form. The children signed a term of assent. This study was conducted in accordance with the ethical precepts stipulated in the Declaration of Helsinki (revised in Fortaleza in 2013). Treatments were performed at the internal and external clinics of the university as well as a private dental office in the city of Montevideo, Uruguay.

### Inclusion criteria

The study included healthy individuals classified as ASA I, children aged 6–12 years, who had at least one permanent first molar affected by MIH grades 3 and 4 (a and b) according to the MIH Treatment Needs Index (TNI)^14^. The participants also had to present a hypersensitivity score of 2 or higher on the visual analog scale (VAS). Only molars that had fractures but no pulp involvement or active caries were included. Additionally, molars with complete eruption of the entire occlusal surface and at least one-third of the vestibular surface were eligible.

### Exclusion criteria

Molars with hypersensitivity caused by carious lesions were excluded to avoid diagnostic confusion with pulpitis. Teeth that had undergone desensitizing treatments within the last three months, except for the use of daily toothpaste, were also excluded. Furthermore, participants with fixed orthodontic appliances, those who experienced discomfort during the hypersensitivity test, or those who were unable to tolerate the procedure were excluded from the study.

### Involvement of patient and public

The design of the study did not involve the patients’ guardians. However, following data analysis, guardians were given the opportunity to attend a meeting to review the results if they wished. The informed consent document assured the confidentiality of the data for each participant.

### Calculation of sample size

The sample size calculation was revised to ensure that the statistical design was aligned with the study objectives, which aim to test the superiority of the proposed treatment compared to the control. The initial calculation, described in the previously published protocol, was based on an effect size of 0.80 as reported by Muniz et al.^10^. However, during the study, it was identified that this estimate did not adequately reflect the variability observed in the literature^15^, particularly considering the design with seven repeated measures. A new calculation was performed using G*Power software (version 3.1.9.7; Heinrich Heine University, Düsseldorf, Germany), considering a power of 80% (β = 0.20), an alpha error of 0.05, and an effect size of 0.352. This resulted in a required sample size of 10 individuals per group. To ensure robustness in the analysis and account for potential additional outcomes and participant dropouts, the sample size was increased by 100%. This adjustment was considered a technical correction necessary to ensure the scientific validity of the results. This transparent disclosure aims to clarify the methodological steps taken to maintain the rigor and integrity of the study.

### Randomization

The participants were randomly allocated to one of the two groups using a randomization site (randomization.com). A researcher, who was not involved in the treatment phase, conducted the randomization process. Sealed envelopes, each containing a number corresponding to the participant’s group allocation, were distributed. The group assignments were only revealed when the sealed envelopes were opened at the time of treatment, ensuring the allocation was concealed until that point.

Group 1 (control group) (n = 24): Participants in this group used fluoride toothpaste with a concentration ≥ 1000 ppm (Parts Per Million) twice per day, received a glass ionomer sealant, and underwent simulated PBM.

Group 2 (study group) (n = 25): Participants in this group used fluoride toothpaste with a concentration ≥ 1000 ppm twice per day, received a glass ionomer sealant, and underwent active PBM.

### Blinding

The study was conducted as a single-blind clinical trial, where patients were unaware of the specific treatment they received. In the control group, the use of the PBM was simulated with a recorded sound to ensure that participants could not distinguish between treatments with and without the use of the PBM.

### Calibration training

Before the study, a calibration training session was conducted to ensure consistency and reliability in the assessments performed by the examiner. The training involved the evaluation of MIH, the Simplified Oral Hygiene Index (OHI-S) by Greene and Vermillion, and the CCC Sealant Evaluation System. Standardized clinical photographs and case scenarios were used to simulate diagnostic conditions. To assess intraexaminer reliability, the same cases were evaluated on two separate occasions, one week apart. Cohen’s Kappa coefficient was calculated separately for each diagnostic step, with values ≥ 0.80 indicating substantial agreement. The results demonstrated high intraexaminer reliability: MIH diagnosis: κ = 0.87; OHI-S index: κ = 0.82; CCC Sealant Evaluation System: κ = 0.85. This process helped minimize variability and enhance the consistency of the clinical evaluations.

### Interventions

The interventions were performed by a single researcher, who administered PBM (simulated or active) and applied the sealant to the corresponding molars. The same examiner performed the pre-procedure and post-procedure assessments. In the first session, PBM was administered, followed by the application of the glass ionomer sealant. PBM was administered again in subsequent sessions after 48 h and 30 days. The participants received oral hygiene instructions—routine brushing twice per day with toothpaste containing ≥ 1000 ppm of fluoride. All participants received a toothbrush and toothpaste to ensure standardization. The aim of this intervention was to assess whether the participants’ brushing technique would improve with desensitization. No change in brushing behavior was expected on the part of those parents who performed brushing on their children.

### PBM protocol

PBM was performed with low-level Therapy XT infrared diode laser (DMC, São Carlos, Brazil) in three sessions: initial session, after 48 h and after one month. The laser was applied to three perpendicular points in contact with the surface on the mesial vestibular and distal cervical faces and in the center of the occlusal face. An energy of 1 J was applied for 10 s at each point. Table 1 displays the PBM parameters.

**Table 1:** PBM parameters.

PBM parameters	
Application method	Contact
Number of points irradiated in each session	3
Number of sessions	3
Wavelength (nm)	808 ± 10
Irradiance (mW/cm^2^)	3571
Spectral width (FWHM)	4.8 ± 2 nm
Operating mode	Continuous
Power (mW)	100
Type of beam	Multi-mode
Beam area (cm^2^)	0.028
Total exposure time per session (s)	30
Radiant exposure (J/cm^2^)	35.7
Energy per point (J)	1
Total energy per session (J)	3

### Sealant application protocol

1. Prophylaxis was performed using a rotary brush and a fluoride-free prophylactic paste (Prophy Paste, Pharmadent, Montevideo, Uruguay).
2. Relative isolation was performed using cotton rolls and suction with a four-handed technique to ensure moisture control.
3. Conditioning was performed using Ketac Conditioner (20% polyacrylic acid, 3M ESPE, St. Paul, MN, USA), which was applied to the enamel surface for 10 s, followed by rinsing with water and gentle air-drying, as recommended by the manufacturer.
4. Ketac Molar Easymix (3M, St. Paul, Minnesota, USA) sealant left in reaction on application surfaces for ten seconds, following by rinsing with abundant water, drying with compressed air in 2–3 brief intervals with oil-free air or drying with cotton swabs.
5. Handling of the material was performed according to the manufacturer’s instructions. The powder and liquid were mixed until homogeneous and then applied to the pits and fissures of the molars.
6. Small quantity of petroleum jelly on finger used to press glass ionomer cement into pits and fissures—digital impression technique.
7. Control of occlusion without receiving pressure for one hour.

### Control group

Brushing with toothpaste with fluoride concentration ≥ 1000 ppm twice per day, glass ionomer sealant and simulated PBM in three sessions: first session, after 48 h and after one month. The children in this group could have access to treatment with active PBM if exhibiting sensitivity at the end of the study.

### Study group

Brushing with toothpaste with fluoride concentration ≥ 1000 ppm twice per day, glass ionomer sealant and active PBM in three sessions: first session, after 48 h and after one month.

Pre-procedure records were taken and brushing instructions were given in the first session in both groups. The pre-procedure records involved the oral hygiene index (OHI-S), record of MIH (for diagnostic purposes) and determination of hypersensitivity (SCASS/VAS). Hypersensitivity was investigated again in the first session after the administration of active or simulated PBM and after the application of the sealant. New records were made 48 h and 30 days after the initial session in both groups. OHI-S was assessed once per session, while SCASS/VAS was evaluated before and after PBM application in each session. The integrity of the sealant was also examined in the final session (30 days).

### Assessments

#### MIH (yes or no)

The diagnosis of MIH was made exclusively according to the criteria of the European Academy of Paediatric Dentistry (EAPD)^16^, which include demarcated opacity, post-eruptive enamel breakdown, atypical restorations, and extractions due to MIH, with defects greater than 1 mm considered. For inclusion criteria, we followed the Molar-Incisor Hypomineralization Treatment Need Index (MIH-TNI) described by Steffen et al.^14^, classifying participants as follows: 0: grade 3 (exhibiting hypersensitivity without defect), 1: grade 4a (exhibiting hypersensitivity and defect, with a subdivision based on the extent of the defect ≤ 1/3), and 2: grade 4b (exhibiting hypersensitivity and defect, with a subdivision based on the extent of the defect from 1/3 to 2/3).

#### Schiff cold air sensitivity scale (SCASS)

The response to the pain stimulus was evaluated using the Schiff Cold Air Sensitivity Scale (SCASS)^17^, with the following classification:

0 = Subject does not respond to air stimulus. 1 = Subject responds to air stimulus but does not request discontinuation of stimulus. 2 = Subject responds to air stimulus and requests discontinuation or moves from stimulus. 3 = Subject responds to air stimulus, considers stimulus to be painful, and requests discontinuation of the stimulus.

#### Visual analog scale (VAS)

The perception of pain in children was assessed using the Wong-Baker Faces Pain Rating Scale. This scale facilitates communication, allowing for a more accurate assessment of the pain reported by the child^18^.

#### Simplified oral hygiene index (OHI-S) (Greene and Vermillion)

The application of the Simplified Oral Hygiene Index (OHI-S) was performed on the buccal surface of the treated molars, based on the analysis of soft plaque deposits^19^. The following classification was used: 0: no deposits or pigmentation, 1: deposits cover less than one-third or presence of pigmentation; 2: deposits cover more than one-third but less than two-thirds, and 3: deposits cover more than two-thirds of the dental surface.

#### Sealant assessment system

The retention of the sealant was evaluated after 1 month using the CCC Sealant Evaluation System^20^. For statistical purposes, the following classification was considered: A: Sealant present on the entire fissure system; B: Sealant present on > 50% of the fissure pattern, but some areas missing; C: Sealant present on < 50% of the fissure pattern; D: No sealant present.

### Statistical analysis

The normality of data distribution and homogeneity of variance were confirmed by Shapiro–Wilk. Continuous variables are described with mean and standard deviation and categorical variables by relative frequency. The chi-square test was used to analyze molar-incisor hypomineralization and the presence of sealants after 30 days. For the analysis of SCASS scale, Visual Analog Scale and Oral Hygiene Index responses over time, a Generalized Estimating Equations model (GEE) was applied, considering two factors (group and time), an unstructured correlation matrix, and a robust covariance matrix with a linear distribution. Post hoc comparisons were performed by Bonferroni’s complementary test to locate differences. Additionally, Spearman’s rank correlation coefficient (*rho*) was used to assess relationships between molar-incisor hypomineralization severity, oral hygiene, hypersensitivity outcomes (SCASS and VAS scores), and sealant durability at 30 days. Statistical analyses and the figure developed were performed using Statistical Package for the Social Sciences (version 20.0, SPSS Inc, Chicago, IL, USA) and GraphPad Prism (GraphPad Software Inc., La Jolla, CA, USA) respectively. For analysis, statistical significance was accepted at *p* < 0.05.

## Results

### General characteristics

Table 2 presents the general characteristics, evaluated teeth distribution and molar-incisor hypomineralization, across the treatment and control groups. Participants had a mean age of 8.5 ± 1.6 years, with no significant difference between groups (*p* = 0.285). The proportion of boys and girls was also similar across groups (*p* = 1.000). Regarding the distribution of evaluated teeth, no significant differences were observed between groups (*p* = 0.203). The percentage of evaluated teeth ranged from 16.0 to 40.0% across tooth types (16, 26, 36, and 46). According to the MIH-TNI classification, there was no significant difference between groups (*p* = 0.118). The distribution across severity grades was as follows: Grade 3 (16.3% overall), Grade 4a (51.0% overall), and Grade 4b (32.7% overall).

**Table 2:** General characteristics, evaluated teeth, presence of hypomineralization in children with MIH.

Variables	All (n = 49)	Treatment (n = 25)	Control (n = 24)	p
Age, years	8.5 ± 1.6	8.8 ± 1.7	8.3 ± 1.6	0.285
Sex, %				1.000
Girls	57.1	56.0	58.3	
Boys	42.9	44.0	41.7	
Evaluated teeth, %				0.203
Tooth 16	28.6	40.0	16.7	
Tooth 26	24.5	24.0	25.0	
Tooth 36	26.5	16.0	37.5	
Tooth 46	20.4	20.0	20.8	
MIH-TNI %				0.118
Grade 3	16.3	8.0	25.0	
Grade 4a	51.0	48.0	54.2	
Grade 4b	32.7	44.0	20.8	

Data are presented as mean ± standard deviation and relative frequency (%).

### SCASS Score responses

**Fig. 2:**
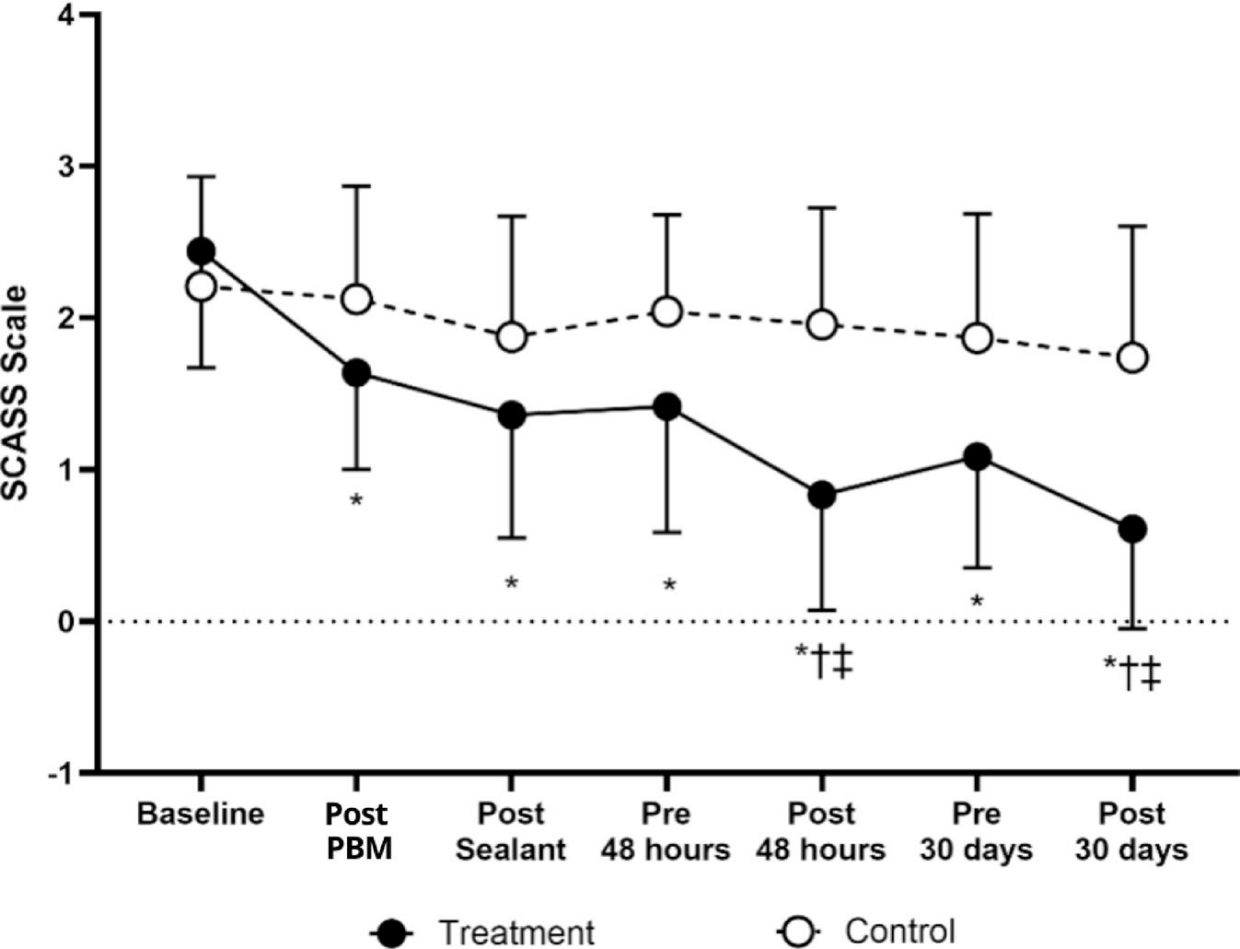
Response of the SCASS scale between groups over time in children with MIH. * = *p* < 0.05 versus baseline; † = *p* < 0.05 versus previous time pont; ‡ = *p* < 0.05 versus control.

Figure 2 presents the SCASS scale responses over time, considering the interaction term in the model. At all time points after baseline, the treatment group exhibited significantly lower values (*p* < 0.05). A significant difference was observed within the treatment group between the pre-48-h and post-48-h assessment of the second PBM application (0.9 ± 1.1 vs. 1.2 ± 1.1, p < 0.001) and between the pre-30-day and post-30-day assessment of the third PBM application (1.2 ± 1.1 vs. 0.7 ± 1.0, p < 0.001). Furthermore, a significant difference was found between the treatment and control groups at the 48-h post-PBM assessment after the second PBM application (treatment: 0.9 ± 1.1 vs. control: 1.9 ± 1.1, *p* < 0.001) and at the 30-day post-PBM assessment after the third PBM application (treatment: 0.7 ± 1.0 vs. control: 1.7 ± 1.2, *p* < 0.001). No other comparisons reached statistical significance.

The treatment group exhibited significantly lower values at all time points after baseline (*p* < 0.05). The GEE model revealed that group allocation was a significant predictor of SCASS scores, with the treatment group showing a significantly lower SCASS score than the control group (B = 0.988, 95% CI 0.541–1.435, *p* < 0.001), and time was also a significant predictor, with SCASS scores decreasing over time (*p* < 0.001 for most time points). A significant interaction between group and time was observed at the 48-h post-PBM assessment, where the treatment group showed a greater reduction in SCASS compared to the control (B = − 1.098, 95% CI − 1.602 to − 0.593, *p* < 0.001). Significant differences were also found in the post-sealant assessment of the first PBM application (B = − 0.486, 95% CI − 0.900 to − 0.071, *p* = 0.022), in the pre-30-day assessment of the third PBM application (B = − 0.526, 95% CI − 0.954 to − 0.098, *p* = 0.016) and in the post-30-day assessment of the third PBM application (B = − 0.376, 95% CI − 0.623 to − 0.129, *p* = 0.003).

### Visual analog scale (VAS) score responses

**Fig. 3:**
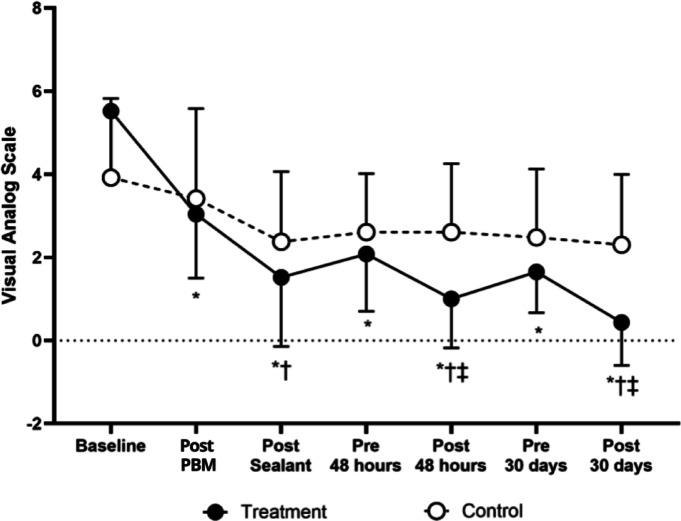
Response of the Visual Analog Scale between groups over time in children with MIH. * = *p* < 0.05 versus baseline; † = *p* < 0.05 versus previous time pont; ‡ = *p* < 0.05 versus control.

Figure 3 presents VAS responses over time, accounting for the interaction term in the model. At all time points after baseline, the treatment group exhibited significantly lower values (*p* < 0.05). A significant difference was observed within the treatment group between the pre-assessment of the second PBM application and the post-48-h assessment (2.1 ± 1.9 vs. 1.0 ± 1.6, *p* < 0.001), as well as between the pre-assessment of the third PBM application and the post-30-day assessment (1.7 ± 1.4 vs. 0.4 ± 1.5, *p* < 0.001). Furthermore, a significant difference was found between the treatment and control groups 48 h after the second PBM application (treatment: 1.0 ± 1.6 vs. control: 2.5 ± 2.4, *p* = 0.040) and 30 days after the third PBM application (treatment: 0.4 ± 1.5 vs. control: 2.1 ± 2.5, *p* = 0.004). No other comparisons reached statistical significance.

The results of the GEE model further confirmed these findings. The interaction effect between treatment allocation and the first post- PBM assessment was significant (B = − 3.341, SE = 0.449, *p* < 0.001), as well as for other post-baseline assessments (*p* < 0.05), except for the pre-assessment of the third application (*p* = 0.433). The main effect of treatment allocation also remained significant (B = 1.709, SE = 0.418, *p* < 0.001), reinforcing the lower overall VAS scores in the treatment group over time.

### Correlation between SCASS and VAS

**Fig. 4:**
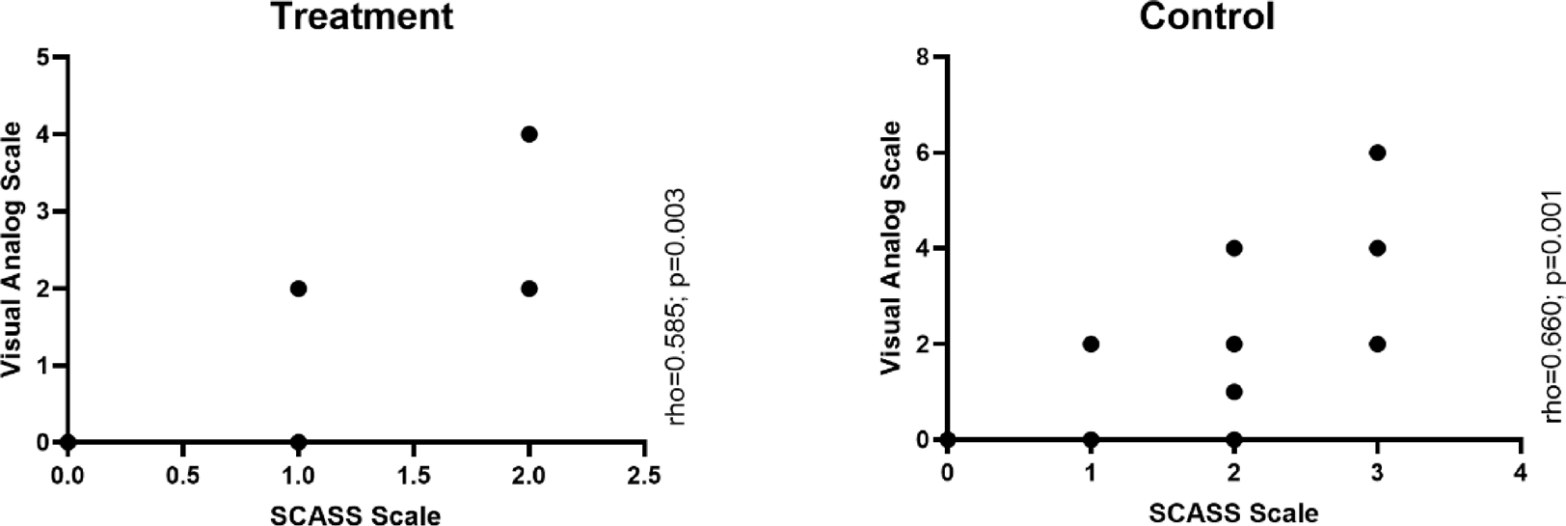
Correlation of the SCASS with Visual Analog Scale between groups in children with MIH.

SCASS and VAS scores exhibited a significant positive correlation in both groups, indicating that higher SCASS scores were associated with higher VAS scores (Fig. 4). In the control group, the correlation was strong (ρ = 0.660, *p* = 0.001), while in the treatment group, a significant association was also observed (ρ = 0.585, *p* = 0.003). These findings suggest that both SCASS and VAS consistently reflect participants’ perceptions of dental hypersensitivity.

### Oral hygiene index score responses

**Fig. 5:**
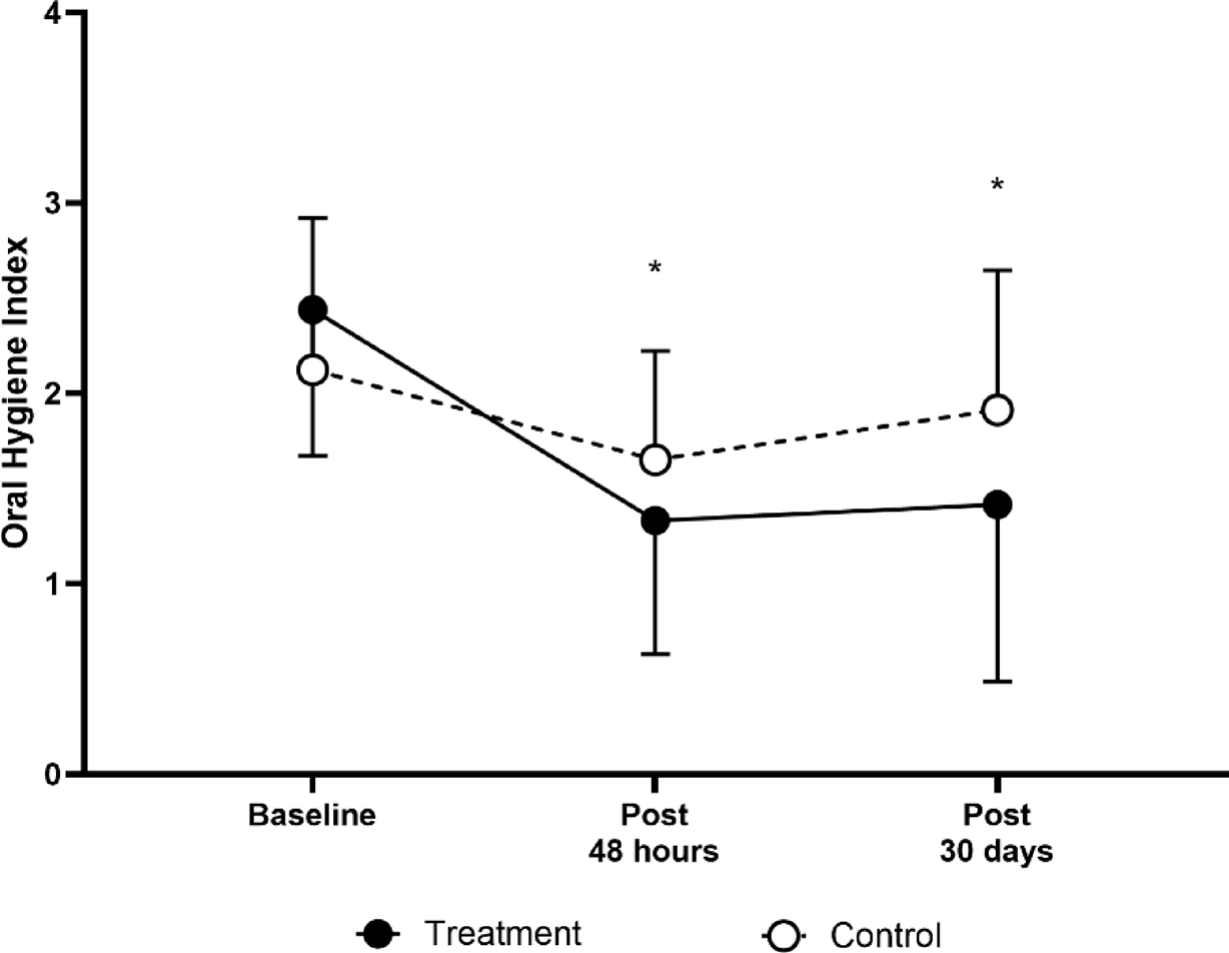
Response of the Oral Hygiene Index between groups over time in children with MIH. * = *p* < 0.05 versus baseline.

Figure 5 presents OHI-S scale responses over time. In the treatment group, significant reductions were observed at the 48-h post- PBM assessment compared to baseline (2.4 ± 1.0 vs. 1.3 ± 1.0, *p* < 0.001) and at the 30-day post-PBM assessment compared to baseline (2.4 ± 1.0 vs. 1.4 ± 1.3, *p* < 0.001). However, no significant differences were found between the treatment and control groups at any time point.

The results of the GEE model indicated a significant main effect of time to first post- PBM assessment (B = − 0.788, SE = 0.199, *p* < 0.001), suggesting an improvement in oral hygiene over time within the treatment group. However, the interaction effects between treatment allocation and subsequent time points were not statistically significant (*p* > 0.05), indicating that the rate of improvement did not differ significantly between groups.

### Correlation between hypersensitivity and oral hygiene

**Fig. 6:**
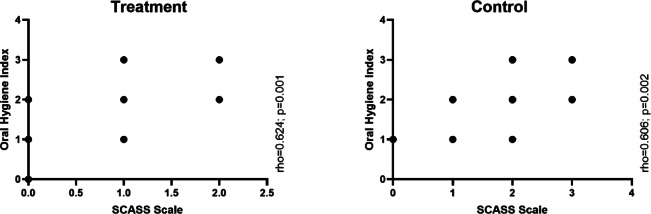
Correlation of the SCASS with Oral Hygiene Index between groups in children with MIH.

As illustrated in Fig. 6, the oral hygiene index showed a strong positive correlation with SCASS scores in both the control group (ρ = 0.606, *p* = 0.002) and the treatment group (ρ = 0.624, *p* = 0.001). This indicates that poorer oral hygiene conditions were associated with higher levels of dental hypersensitivity over time.

Table 3 shows the Sealant application in children with MIH. Sealant presence differed significantly between groups (*p* = 0.002). In the treatment group, 56.5% of teeth had sealant present in all grooves, while this percentage was lower in the control group (17.4%). Conversely, the absence of sealant was reported in 17.4% of control group teeth and none in the treatment group.

**Table 3:** Sealant application in children with MIH.

Variables	All (n = 49)	Treatment (n = 25)	Control (n = 24)	*p*
Sealant presence, %				0.002
Present on entire fissure system	37.0	56.5	17.4	
Present on > 50% of fissure pattern, some missing	26.1	30.4	21.7	
Present on < 50% of fissure pattern	28.3	13.0	43.5	
No sealant present	8.7	0.0	17.4	

Data are presented as mean ± standard deviation and relative frequency (%).

### Correlation between hypersensitivity and sealant

**Fig. 7:**
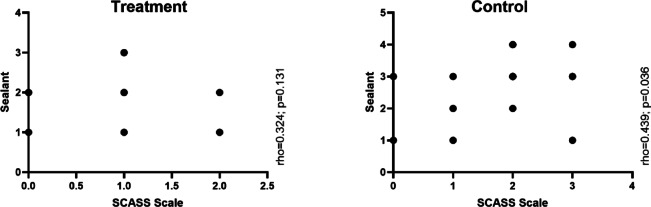
Correlation of the SCASS with sealant between groups in children with MIH.

As shown in Fig. 7, sealant durability exhibited a significant positive correlation with hypersensitivity, as assessed by the SCASS scale. In the control group, a moderate correlation was observed (ρ = 0.439, *p* = 0.036), Indicating that lower sealant durability was associated with higher levels of hypersensitivity. In the treatment group, this correlation was not significant (ρ = 0.324, *p* = 0.131).

Additional clinical outcomes, including SCASS scale scores, VAS and OHI over time, are detailed in the Supplementary Table S1. These data compare responses between groups and provide further insight into the clinical progression and treatment effects in children with MIH.

## Discussion

Hypersensitivity in molars with MIH is the result of the porosity of the enamel, which enables the penetration of bacteria into the dental tissue. Bacterial infiltration triggers subclinical pulpal inflammation, which is considered the main cause of this symptom, along with post-eruptive fracture^21^.

Individuals with hypersensitivity reduce brushing in the affected area to avoid worsening discomfort. This hypersensitivity can also lead to a decline in dental function, limiting the natural self-cleaning of the oral cavity through the action of the tongue, cheeks, and saliva. As a result, biofilm accumulation increases and, combined with enamel porosity, increases the risk of caries^5^. Caries in teeth with MIH exhibit an atypical pattern in terms of location and rate of progression, often occurring in patients who would normally be considered at low risk for caries^22^.

In practice, hypersensitivity also constitutes a problem with regards to the treatment of these teeth, as frequent pain exerts an impact on the patient’s behavior, making clinical management difficult^18^. Compressed air from the three-way syringe used for inspection can cause acute pain, which complicates the behavior management of the patient. Problems with adequate analgesia are also described^5^.

Once MIH with hypersensitivity is diagnosed, treatment should prioritize alleviating hypersensitivity before considering the most appropriate long-term treatment strategy. Various treatment approaches have been tested to reduce hypersensitivity in patients with MIH, with positive results in most cases^23^. The combination of neural action through reversible blocking of the conduction of central and peripheral sensory nerve fibers and the release of the neurotransmitter beta-endorphin, which promotes analgesia, along with dentinal tubule obliteration, offers greater benefits for teeth affected by MIH^10^.

The literature clearly establishes the efficacy of sealants in treating hypersensitivity in teeth with MIH^11^. Furthermore, a previous study demonstrated that combining therapeutic approaches, such as neural and obliterative actions, can be more beneficial than isolated treatments^10^. In this context, our hypothesis that the combination of sealant with PBM could improve the assessed indices, such as hypersensitivity, oral hygiene index, and sealant retention, is confirmed by the results of this study, highlighting an additional benefit from the use of PBM. This knowledge is particularly relevant as it emphasizes the importance of initial treatment in managing hypersensitivity, which is essential for ensuring the long-term clinical success of restorative approaches.

In the present study, the results demonstrated that the PBM combined with sealant treatment provided a progressive and clinically significant improvement in hypersensitivity symptoms, as assessed by the SCASS scale. After the first PBM application, there was an immediate reduction in hypersensitivity, which became even more pronounced following the sealant application, with an average reduction of 0.5 points on the SCASS scale, corresponding to a 20% decrease in symptoms. This effect was further enhanced 48 h later, with the second PBM application, when the reduction reached 1 point, representing a 50% improvement compared to the control group. In the 30-day pre-evaluation before the third PBM application, the difference between groups remained at 0.5 points, indicating a 25% greater improvement in the treatment group. This effect became even more evident after the third PBM session, with an additional reduction of 0.4 points, equating to a 20–25% decrease in symptoms compared to the control group. These findings emphasize the clinical benefit of combining PBM with sealant for reducing hypersensitivity in MIH-affected teeth.

On the VAS scale, the treatment group initially experienced a 0.5-point reduction in discomfort immediately after the first session, which increased to 1 point after the second PBM application, representing a 50% decrease in perceived hypersensitivity compared to the control group. This improvement became even more pronounced after the third session, with an average reduction of 1.3 points, corresponding to a 65% decrease in reported discomfort.

Furthermore, the correlation between the VAS and SCASS scales suggests that both consistently reflect patients’ perceptions of hypersensitivity reduction, reinforcing the reliability of these measures in evaluating treatment efficacy. Overall, these findings support the clinical superiority of the combined PBM and sealant protocol over the use of sealant alone, offering a more effective and long-lasting approach to managing hypersensitivity in teeth with MIH.

The findings of the present study are supported by previous research demonstrating the immediate desensitizing effect of laser therapy using the same parameters^10^. Furthermore, the potential of combined therapies, integrating the neural action of PBM with the obliterative effect of casein phosphopeptide-amorphous calcium phosphate fluoride (CPP-ACPF) mousse, has been previously reported, showing superior clinical outcomes^15^.

When comparing the findings of the present study with those of a recent investigation^24^ that evaluated the effectiveness of glass ionomer sealants in molars affected by MIH, some important differences were identified. Although both studies used similar materials, such as glass ionomer sealants, the results regarding hypersensitivity reduction and sealant retention varied substantially. In the referenced study, although the main treatment consisted of sealant application, participants were instructed to brush their teeth daily with fluoride toothpaste and to use a high-concentration fluoride gel weekly (12,500 ppm), which may have contributed to the reduction in hypersensitivity over time. Regarding sealant retention, the study reported a 7.2% loss over 12 weeks, whereas in the present study, the control group exhibited a greater loss in just 30 days. In contrast, the group treated with PBM showed no loss of sealant after 30 days. These findings suggest that PBM may play a relevant role in maintaining the sealant, possibly by reducing hypersensitivity and improving the material’s adhesion to enamel. Moreover, a correlation was found between hypersensitivity and sealant retention, particularly in the control group, where higher levels of hypersensitivity were associated with lower retention rates, reinforcing the importance of therapeutic strategies that mitigate hypersensitivity to enhance sealant durability.

As hypersensitivity intensity decreased, a tendency toward improved oral hygiene was observed. To investigate this relationship, the Simplified Oral Hygiene Index (OHI-S) was used, and a significant reduction in scores was noted in both groups.

In the treatment group, the OHI-S score decreased by an average of approximately 1.1 points after 48 h, representing a 45% reduction in oral hygiene index scores, an effect that was maintained at the 30-day evaluation. Additionally, a strong positive correlation was found between OHI-S and SCASS scores, suggesting that poorer oral hygiene conditions were associated with higher levels of dental hypersensitivity over time. The relationship between the intensification of hypersensitivity and the deterioration of oral hygiene has previously been reported^25^ and was confirmed in the present investigation.

The administration of PBM and the consequent reduction in hypersensitivity may contribute to improved oral hygiene in children with MIH. This is a significant finding, as better hygiene practices can help reduce the risk of caries in affected teeth. Furthermore, this study confirmed the greater durability of the glass ionomer sealant in the treatment group, which may be attributed to the decrease in hypersensitivity, allowing for more effective brushing. Clinically, the improved sealant retention observed in the PBM group suggests that incorporating PBM could enhance the long-term effectiveness of sealants in children with MIH. This approach may reduce the frequency of reapplications and improve patient comfort by addressing hypersensitivity. Notably, a strong positive correlation was observed in the control group between sealant durability and hypersensitivity, further supporting these findings. Future studies should investigate the impact of PBM on the tooth surface and its influence on the adherence of materials.

Among the strengths of this study, the methodological aspects stand out, as they enhance the reliability and clinical applicability of the findings. A rigorously standardized intervention protocol was implemented, with all procedures performed by a single experienced and calibrated operator, ensuring consistency in the applied methods. However, some limitations should be acknowledged. Although the age range of participants (6–12 years) could theoretically influence hypersensitivity outcomes, since younger children tended to present slightly higher SCASS scores^26^, no statistically significant age difference was found between groups (*p* = 0.285), and the age distribution was relatively homogeneous. Additionally, the use of Generalized Estimating Equations (GEE) in our analysis helped account for interindividual variability over time, reducing the potential impact of age as a confounding factor. Furthermore, the lack of blinded outcome assessors and the short follow-up period represent additional limitations. Although the 30-day follow-up was sufficient to detect significant correlations between hypersensitivity, oral hygiene, and sealant retention, longer-term studies are needed to assess the sustainability of these effects over time.

## Conclusion

The administration of PBM combined with glass ionomer cement reduced hypersensitivity, leading to improved oral hygiene in molars with MIH. The reduction in hypersensitivity increased the sealant’s durability in MIH-affected teeth. Additionally, a significant decrease was observed in the SCASS and VAS scales after PBM, demonstrating its potential use in improving hypersensitivity.

## Supplementary Information

Below is the link to the electronic supplementary material.

Supplementary Material 1

## Data Availability

All data generated or analysed during this study are included in this published article.
