# Supplementary material for: Randomized clinical trial of photobiomodulation and glass ionomer sealant for hypersensitivity in molar incisor hypomineralization
Source: Sci Rep. 2025 Aug 29;15:31911. doi: 10.1038/s41598-025-17454-8 (PMC12397276; doi:10.1038/s41598-025-17454-8)
Supplement: Supplementary file 1 — Supplementary Material 1 [file 41598_2025_17454_MOESM1_ESM.docx]

**Supplementary Table S1** Response of the SCASS scale, Visual Analog Scale (VAS), and Oral Hygiene Index (OHI) between groups over time in children with Molar Incisor Hypomineralization (n = 49).

|  | | | | | | | | | | |
| --- | --- | --- | --- | --- | --- | --- | --- | --- | --- | --- |
|  |  | 1^st^ PBM session | | 2^nd^ PBM session | | 3^rd^ PBM session | |  |  |  |
|  | Baseline | Post  PBM | Post  Sealant | Pre  48 hours | Post  48 hours | Pre  30 days | Post  30 days | group | time | interaction |
| SCASS scale | | |  |  |  |  |  | <0.001 | 0.000 | <0.001 |
| Treatment | 2.3±1.0 | 1.6±0.8* | 1.4±1.1* | 1.4±1.2* | 0.9±1.1***†**‡ | 1.2±1.1* | 0.7±1.0***†**‡ |  |  |  |
| Control | 2.2±1.0 | 2.1±1.0 | 1.9±1.1 | 2.0±0.9 | 1.9±1.1 | 1.8±1.2 | 1.7±1.2 |  |  |  |
| Visual Analog Scale | | |  |  |  |  |  | 0.157 | 0.000 | <0.001 |
| Treatment | 5.5±2.3 | 3.1±2.2* | 1.6±2.4*† | 2.1±1.9* | 1.0±1.6*†‡ | 1.7±1.4* | 0.4±1.5*†‡ |  |  |  |
| Control | 4.0±2.7 | 3.4±3.0 | 2.4±2.4*† | 2.5±2.1* | 2.5±2.4* | 2.3±2.4* | 2.1±2.5* |  |  |  |
| Oral Hygiene Index | | |  |  |  |  |  | 0.359 | <0.001 | <0.001 |
| Treatment | 2.4±1.0 | - | - | - | 1.3±1.0* | - | 1.4±1.3* |  |  |  |
| Control | 2.1±1.1 | - | - | - | 1.6±0.8 | - | 1.9±1.0 |  |  |  |
| * = p<0.05 vs baseline; †= p<0.05 vs previous time point; ‡= p<0.05 vs control | | | | | | | | | | |
